# Seedling emergence and biomass production of soybean cultivars under wheat-soybean relay cropping

**DOI:** 10.1371/journal.pone.0293671

**Published:** 2023-11-01

**Authors:** Jay Ram Lamichhane, Carla Varaillas, Philippe Debaeke

**Affiliations:** INRAE, University of Toulouse, UMR AGIR, Castanet-Tolosan, France; Central Research Institute for Dryland Agriculture, INDIA

## Abstract

Diversification and intensification of cropping systems can ensure farm profitability while reducing negative environmental impacts of agriculture. Wheat-soybean relay cropping (RC), which consists in planting soybean into standing wheat prior to its harvest, may have this potential although it is poorly adopted by French and European farmers. One of the reasons underlying this lack of adoption could be poor emergence rates and biomass production of soybean, due to a severe competition from the already established primary crop for water, light and nutrients during the co-growth or intercrop phase. All these constraints during the early plant growth could finally affect soybean grain yield and thus farm profitability. Here, we performed a laboratory experiment followed by a 2-year field trial (2021–2022) to investigate potential differences among seven soybean cultivars belonging to different maturity groups (from very early to late) in terms of early growth traits viz. seed germination, seedling emergence vigor and final rates, and early biomass production in wheat-soybean RC. A reference soybean variety belonging to late maturity group (cv. ES Pallador) was also sown under conventional cropping system as control treatment (hereafter referred to as CC). Under laboratory conditions, the base water potential for germination ranged from -0.65 to -0.45 MPa with significant differences (p<0.001) among the tested cultivars indicating their differential tolerance to water stress. Under field conditions, seedling emergence vigor, an index explaining the speed of emergence, ranged from 0.23 to 0.41 and from 0.24 to 0.33 while final emergence rates ranged from 69% to 93% and from 65 to 90% in 2021 and 2022, respectively. We found significant effect of cultivar, year and cultivar x year interaction on emergence vigor (p<0.001) and final emergence rates (p<0.01, p<0.05 and p<0.01, respectively) of soybean cultivars. Significantly higher emergence vigor of the referent cv. ES Pallador was observed in RC compared to CC cropping system in 2021 (0.40 and 0.34, respectively) but not in 2022 (0.29 and 0.31, respectively). Water stress in the seedbed was higher in RC compared to the CC and was the main cause affecting seed germination and seedling emergence vigor especially in 2022. We found a positive correlation between seedling emergence vigor and seedling final emergence rates indicating that a lower speed of seedling emergence, due to seedbed stress factors, affects final emergence rates of soybean. Post-emergence losses due to pigeons were significantly higher (p<0.001) in CC compared to RC (30% and 2% in 2021, and 29% and 2% in 2022 in CC and RC, respectively). Significantly higher biomass production was observed in CC compared to that in RC both in 2021 (162 vs 33 g/m^2^ of dry matter; p<0.001) and 2022 (252 vs 60 g/m^2^ of dry matter; p<0.001). Overall, pre-/post-emergence water stress in the seedbed and post-emergence damage due to pigeons are the most important factors affecting a uniform and robust soybean establishment under RC and CC, respectively under southern French conditions.

## 1. Introduction

Relay cropping (RC) is a multiple cropping method that consists in planting a second crop (known as relay crop) into standing first crop (known as primary crop) prior to the primary crop harvest thereby allowing farmers to grow two crops within the same year [1, 2]. RC provides several advantages including higher land equivalent ratio compared to the intercropping system with two crops growing simultaneously due to an increased temporal niche differentiation [3–5]. In addition, RC represents an alternative to sequential double cropping (i.e. planting a second crop after the harvest of the first crop in the same year) in terms of harvesting two crops per year when the latter is not feasible due to unfavourable weather conditions (insufficient growing degree days, risks of frequent rainfalls and higher air moisture in the autumn affecting the maturity phase of late-sown crops), especially across temperate regions [6].

Small-grain cereals-legumes RC has been reported to provide numerous benefits including reduced soil nitrogen loss [7], greater land equivalent ratio [8], better weed control [9, 10] and higher grain yield [8, 9, 11, 12] compared to sole cropping systems. In France, most of the studies performed on small-grain cereals-legumes RC were on winter wheat-forage legumes [13, 14] or winter wheat-legume cover crops [7, 15]. In these RC systems, legume species were not planted for grain harvest but rather for biomass production purpose. However, the lengthening of the growing season across the northern and central Europe due to global warming make it possible to practice RC of wheat-grain legumes. This is particularly the case for wheat-soybean RC thanks to the availability of a large number of commercial soybean cultivars from MG 000 to II on the European market [16]. Inclusion of soybean in the RC system in France and Europe not only allows for spatiotemporal diversification of cropping systems but also contributes to protein self-sufficiency, and farm profitability by allowing the harvest of two crops within the same year. Despite all these potential benefits, very limited knowledge is available in the scientific literature on wheat-soybean RC from France or Europe. Indeed, a recent study highlighted a very limited adoption of relay cropping in France compared to sequential double cropping [17].

Previous studies focusing on RC systems showed that the relay crop planted into a standing primary crop suffers from poor emergence due to competition for soil moisture [13, 18–22]. Poor competition ability of the relay crop for soil moisture with an already standing primary crop may thus strongly limit a uniform and robust establishment of the relay crop. More specifically to soybean, the establishment of the crop could face major challenges under RC as soybean is highly sensitive to water stress compared to many other field crops as shown by its high base water potential values for germination [23, 24]. Therefore, knowledge about how soybean will respond under wheat-soybean RC in general and during the competition phase (also known as co-growth or intercrop phase) in particular may allow to choose the most adapted soybean genotypes for RC. In light of this, we aimed at analyzing the potential genetic variability for early growth traits (i.e. seed germination, seedling emergence and biomass production) of seven soybean cultivars belonging to different maturity groups (MGs) under wheat-soybean RC. We only focus on the competition phase as a successful crop establishment and biomass production at the end of the competition phase is critical for the relay crop productivity.

The specific objectives of this study were to: i) analyze potential differences in germination, emergence and biomass production of seven soybean cultivars under wheat-soybean RC system; ii) compare whether seedbed conditions under wheat-soybean RC are different than those under sole cropping of soybean planted under conventional cropping system (hereafter referred to as CC); and iii) quantify emergence losses, post-emergence damage and identify their causes both under RC and CC. For the purpose of comparison, a soybean cultivar (ES Pallador) was included in the study as control treatment under CC as commonly practiced by growers in France.

## 2. Materials and methods

### 2.1. Laboratory experiments to determine the base water potential for germination of seven soybean cultivars

The base water potential value for germination (Ψ_b_) is an important indicator of water stress tolerance of a given plant [25]. The knowledge of Ψ_b_ for any species or varieties is thus critical for decision making especially when performing experiments across environmental conditions characterized by frequent water shortage in the seedbed as is the case under RC. Therefore, laboratory experiments were performed to determine the Ψ_b_ of seven soybean cultivars belonging to different MGs **(Table 1)**.

**Table 1:** Key characteristics of seven soybean cultivars tested under laboratory and field conditions in 2021 and 2022.

Cultivar	Maturity group	Breeder	TSM (g)	Germination capacity (%)^a^
RGT SIGMA	000	RAGT 2N	187	98 ± 1
RGT SPHINXA	000	RAGT 2N	211	99^a^ ± 0
RGT STUMPA	00	RAGT 2N	176	95 ± 1
ES TRIBOR	0	EURALIS SEMENCES	199	99 ± 0
RGT SPEEDA	0	RAGT 2N	223	99 ± 0
ES ISIDOR	I	EURALIS SEMENCES	228	98 ± 0
ES PALLADOR*	I	EURALIS SEMENCES	181	96 ± 1

TSM: Thousand Seed Mass; 000: Very Early, 00: Early; 0: Medium Early; I: Late

*Standard variety used as reference

^a^Germination capacity was determined using a standard germination test

Germination was tested under four different water potentials (0, -0.10, -0.25, 0.50, and -0.75 MPa) to simulate the effect of different water stress levels on seed germination [26]. A non-treated commercial seed lot of the cultivars, which was produced under standard growing conditions in France, was used. Because the use of polyethylene glycol (PEG) solution delays seed germination (and increases the length of incubation) with an increased risk of seed contamination by mold, the seeds were disinfected by using 1% Sodium hypochlorite solution for 10 min followed by two rinses in distilled water. For each cultivar, four replicates, each of 25 disinfected seeds, were laid onto flat Whatman® filter paper in 90 mm Petri dishes with 20 ml of osmotic solutions of high molecular weight PEG (Polyethylene glycol 8000, ref. SIGMA 25322-68-3). The PEG 8000 concentrations 0, 73, 130, 195, and 245 g/l of deionized water were used to obtain the water potentials of 0, -0.10, -0.25, 0.50, and 0.75 MPa, respectively [27, 28]. The quantity of PEG solution used was higher (20 ml) than the quantity of deionized water used (8 ml) since time to reach the maximum germination is longer with PEG solution. The dishes were incubated at 20 ±1°C and temperatures were hourly recorded with sensors. Seed germination was assessed up to three times per day until no further germination was observed. A given seed was considered germinated when the radicle was>3 mm. At each observation date, the germinated seeds were removed from the dishes. The Ψ_b_ values were determined by fitting a Gompertz function to the observed germination rates as this function has been widely used in various facets of biology [29].

### 2.2. Field experiments

#### 2.2.1. Experimental site, seedbed preparation, and sowing operations

Field experiments were carried out for two consecutive years 2021–2022 in Auzeville experimental station of INRAE (43.53°N, 1.58°E), in Southwestern France. Key information on soil physico-chemical characteristics and sowing conditions of the study sites are summarized in **Table 2**.

**Table 2:** Final average rate of seed germination (± standard deviation) of seven soybean cultivars tested under laboratory conditions under a wide range of water potentials.

Cultivar	0 Mpa	-0.1 Mpa	-0.25 Mpa	-0.5 Mpa	Ψb
RGT SIGMA	97^a^ ± 1	99^a^ ± 0	99^b^ ± 0	99 ^b^ ± 0	-0.52^cd^
RGT SPHINXA	99 ^a^ ± 0	99 ^a^ ± 0	91 ^ab^ ± 0	97 ^b^ ± 0	-0.52^cd^
RGT STUMPA	95 ^a^ ± 1	100 ^a^ ± 0	97 ^b^ ± 1	92 ^b^ ± 2	-0.57^ac^
ES TRIBOR	99 ^a^ ± 0	100 ^a^ ± 0	100 ^b^ ± 0	95 ^b^ ± 4	-0.53 ^bcd^
RGT SPEEDA	99 ^a^ ± 0	100 ^a^ ± 0	100 ^b^ ± 0	99 ^a^ ± 0	-0.45^d^
ES ISIDOR	98 ^a^ ± 0	94 ^a^ ± 0	81^a^ ± 2	23 ^a^ ± 13	-0.64^ab^
ES PALLADOR	96 ^a^ ± 1	96 ^a^ ± 1	94 ^b^ ± 1	84 ^b^ ± 16	-0.65^a^
**Mean**	**98 ± 3**	**98 ± 3**	**95 ± 6**	**84 ± 19**	**-0.55 ± 0.07**
	**ns**	*	***	***	***

Means followed by the same letter are not significantly different at p < 0.05

*p<0.05

***p<0.001; ns: not significant

We selected wheat-soybean RC as preliminary results showed a good potential of this system across the study site [30]. Following a 9-cm stubble tillage (Agrisem), wheat was sown using seed driller (Nodet) in two-row strips with a spacing of 15 cm between rows and 35 cm between the strips to ensure 50 cm spacing between the future rows of soybean in RC. While we planted the same soybean cultivars used for laboratory experiments **(Table 1)** in the field for both years, we used soft wheat cv. Izalco in the first year and durum wheat cv. RGT Voilur in the second year (**Table 2).** The soybean cultivars were planted into the standing wheat almost at the heading stage (between stage 47 and 51 of the BBCH scale). Soybean was planted in 6 x 12 m micro-plots (three microplots/cultivar in total for each year) that were arranged under completely randomized blocks. In addition to RC, cv. ES Pallador was also sown under conventional cropping system as control treatment (hereafter referred to as CC), as generally practiced by growers in the area. For CC, tillage was performed with 4-body plough at 30 cm depth by the end of autumn and a 9-cm stubble tillage was followed with seedbed cultivator (Kongskilde, tractor New Holland 115) to prepare the seedbed prior to sowing cv. ES Pallador. A precision seeder (Maxima-Khun, Claas Arion 410 150cm; 95 hp) was used to plant soybean at a 50 cm spacing between the rows both under RC and CC. No protection measures of the experimental plots were applied to allow potential seed or seedling damage due to animal pests (especially pigeons). All the crop management interventions conducted over the experimental duration are reported in **Table 3**.

**Table 3:** Seedbed and sowing conditions of seven soybean cultivars for two consecutive years 2021–2022 at the Auzeville experimental station.

Key characteristics	Experimental year	
	2020–2021	2022
**Soil Granulometry**		
Clay (g.g-1)	0.36	0.32
Silt (g.g-1)	0.34	0.32
Sand (g.g-1)	0.31	0.36
**Soil chemical characteristics**		
Total Carbon (g.g-1)	0.0132	0.0134
Total Nitrogen (g.g-1)	0.00076	0.00088
C/N ratio	8.9	8.79
pH	7.7	7.2
Organic matter (g.g-1)	0.0108	0.0135
**Characteristics of the primary crop**		
Type	Soft wheat cv. Izalco	Durum wheat cv. RGT Voilur
Sowing date	10 November 2020	3 February 2022
Harvest date	8 July 2021	6 July 2022
Sowing density (kg.ha^-1^)	160	120
Sowing depth (cm)	3	3
**Nitrogen fertilization of the primary crop**		
Nitrogen (kg.ha^-1^)	59 (15 February 2021), 76 (8 April 2021)	79 (7 March 2022)
Nitrogen (kg.ha^-1^)		
**Pesticide treatments**		
Picotop (L.ha^-1^)	1.33 (1 March 2021)	
Elatus Era (L.ha^-1^)	1 (30 April 2021)	
Sticman + Soleil (L.ha^-1^)	0.15 + 1 (10 May 2021)	
Mavrick Flo (L.ha^-1^)	0.2 (21 May 2021)	
Irrigation before soybean sowing (mm)	42 (15 April) 2021	0
**Characteristics of soybean crop**		
Sowing date	6 May 2021	9 May 2022
Plot size (m^2^)	3 m x 12 m	3 m x 12 m
Inter-row distance (cm)	50	50
Sowing density	54	54
Sowing depth	3	3
Irrigation during the competition phase (mm)	28 (15 June 2021)	38 (12 May 2022), 49 (1 June 2022), 36 (16 June 2022)
Duration of the competition phase (Days)	63 (8 July 2021)	71 (16 July 2022)

#### 2.2.2. Climatic data

Soil temperature and water content were recorded using climate sensors (ECH O 5TM, METER Group, Inc. USA). For both experimental years, the sensors were installed in the seedbed of RC and control treatment immediately after sowing. The sensors were installed at three soil depths (3 sensors/depth/block at -3 cm, -5 cm and -10 cm). These sensors delivered hourly temperatures, measured by an onboard thermistor, along with accurate volumetric water content. Data were recorded from sowing to the completion of crop emergence. In addition, climate data (average air temperature and rainfall) obtained from an automatic meteorological station installed at the experimental site were retrieved.

**Fig 1:**
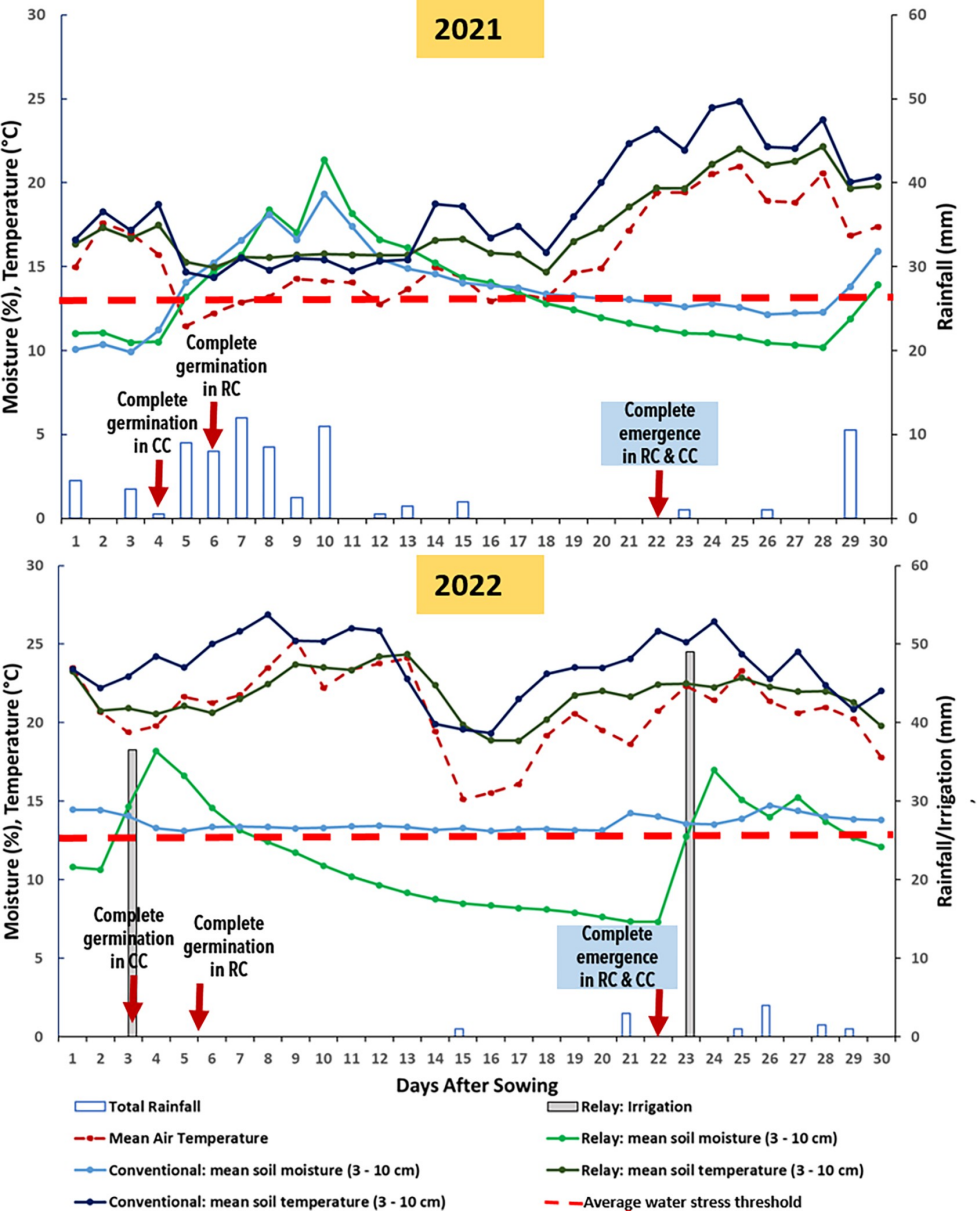
Dynamics of the seedbed soil temperatures, soil moistures, air temperatures and rainfalls at the study site from sowing until crop emergence in 2021 (a) and 2022 (b) under relay and conventional cropping systems at the Auzeville experimental station. Soybean was sown on 6 May and 9 May in 2021 and 2022, respectively. The dotted horizontal red line represents the water stress threshold for soybean germination.

Mean values of daily air temperatures, daily cumulated rainfalls, as well as mean temperatures and gravimetric water content at 0–10 cm soil depth from sowing until the complete emergence for the 2-year experiment are reported in **Fig 1**. In both years, irrigation was applied in RC plots during the competition phase but with many differences in terms of timing and quantity of water applied in relation with rainfalls and evaporative demand. In 2021, no irrigation was applied until soybean emergence and only one irrigation application (28 mm) was made before wheat harvest **(Table 3)**. In contrast, in 2022, three irrigation applications were made: a first 3 days after sowing (DAS; 38 mm), a second (49 mm) just after the completion of emergence, and a third (36 mm) before wheat harvest **(Table 3)**. In contrast, no irrigations were applied in CC. Average air temperature from sowing until emergence was 14±1°C and 22±2°C in 2021 and 2022, respectively. The values of cumulative rainfalls from sowing until emergence were 76 mm and 4 mm, in 2021 and 2022, respectively. Average seedbed temperatures at 0–10 cm soil depth was 14±2°C and 16±1°C in 2021 and 22±1°C and 24±2°C in 2022 in RC and CC, respectively. Average gravimetric water content at 0–10 cm soil depth was 14±3% and 14±2% in 2021 and 11±5% and 13±5% in 2022 in RC and CC, respectively.

Water stress threshold in the seedbed for soybean germination was determined by fitting the relationship between soil water content and water potential to the equation of van Genuchten [31]. To this aim, we used previously available data from the experimental site on soil water holding capacity and Ψ_b_ of soybean varieties as determined in the laboratory experiments. The mean water stress threshold for soybean germination was 13 and 12.5% of gravimetric water content in 2021 and 2022, respectively **(Fig 1)**.

#### 2.2.3. Sowing depth and seed germination measurements

Sowing depth and seed germination measurements require a semi-destructive measurement and therefore they were performed only for cv. ES Pallador both in RC and CC. The sowing depth was determined the day after sowing by making a micro-profile and moving vertically until reaching seeds along the row. Once seeds were retrieved, the sowing depth (i.e. the distance between the seed and soil surface) was measured by using a ruler. The measurement was performed on 5 seeds/row and 4 rows in diagonal /micro-plot for a total of 60 seeds/cultivar in RC and the same number in CC.

The number of germinated seeds was counted every day beginning from 1 DAS. The same method, as for sowing depth, was used on the same number of seeds. Seeds were considered to have germinated successfully if the radicle was > 0.5 cm in length. The measurements were continued until reaching a plateau (the same number of germinated seeds for three consecutive days) and when no rainfall was foreseen in the coming days as rainfall triggers germination of those seeds, which did not germinate due to water stress in the seedbed that is typical across our study sites.

#### 2.2.4. Seedling emergence dynamics and seedling vigor

Seedling emergence dynamics were measured for all the cultivars in RC and for cv. ES Pallador in CC by counting the emerged seedlings in the 12 m^2^ area delimited with plastic pegs for each cultivar (i.e. 2 linear meters/row, 4 rows in diagonal/micro-plot). A seedling was considered emerged when cotyledons were clearly visible over the soil surface [32]. The counting was continued from the beginning of emergence until reaching a plateau with no new emerging seedlings during several days. Time to 50% emergence (T_50_) was determined by fitting a Gompertz function to the observed emergence rates as the T_50_ value is an indicator of rapid crop emergence.

The knowledge on the initial emergence vigor is an important indicator for a rapid, uniform and robust crop establishment across diverse environmental conditions [33–35]. Seedling emergence vigor index (SEVI) ranges from 0.1 to 1 with 0.1 indicating a very slow and 1 indicating rapid emergence. The number of emerged seedlings counted at each observations were used to calculate SEVI using the following formula [36].

SEVI = nes/nts*dc_*1*_ + nes/ nts*dc_*2*_ + ……………+nes/ nts*dc_*n*_

Where *nes* and *nts* indicate the number of emerged and total seedlings, respectively while *dc*_*1*_, *dc*_*2*_ and *dc*_*n*_ indicate days to first, second and final count, respectively.

#### 2.2.5. Causes of non-emergence

The causes of non-emergence were identified for all the cultivars in RC and for cv. ES Pallador in control treatment using a visual diagnostic key. Each symptoms or characteristics leading to emergence losses observed in the field was assigned to a specific stress as follows: i) no seed found: predation of seeds by birds, ii) empty seed coat: damage caused by granivorous, iii) intact seed without necrosis/rotting (N/R): pre-germination abiotic (water or temperature) stress, iv) seed or seedling showing N/R: pre-emergence damping-off, v) presence of holes or larvae in or around seeds/seedling: soil pests (seed maggot, wireworm etc.), vi) seeds germinated, no N/R, no aggregate above, but presence of soil crust: stress due to soil crust, vii) seeds germinated, no N/R, no soil crust but presence of clods above the seedling: seedling blocked under soil clods, stones or crop residues, and viii) seeds germinated, drying seedling, no N/R, no soil crust or aggregates, no or little rainfall after sowing: post-germination water stress.

To this objective, 60 empty points/plot without seedling emergence (20/micro-plot) were randomly selected. A micro-profile was made by a spoon at each empty point moving vertically until reaching non-germinated seeds or non-emerged seedlings. Once seeds or seedling parts were found, their status was annotated according to their characteristics and possible causes of non-emergence were noted.

To calculate rates of germinated seeds and emerged seedlings, the number of germinated seeds and emerged seedlings were converted into percentage for each observations.

#### 2.2.6. Post-emergence damages

Post-emergence seedling damage has been reported as the most important factor affecting soybean establishment when sown as sole crop in many parts of the world including across the study sites [37, 38]. Therefore, the counting of this damage was performed for all the cultivars in RC and for cv. ES Pallador in control treatment. At each observation, the total number of emerged seedlings with or without damage was counted. For damaged seedlings, the type of damage (i.e. partial-damage of cotyledons, total damage due to uprooting of seedlings, or chewed seedlings or seedling parts) was noted. The countings initiated when cotyledons were nearly at the VE stage (i. e. when cotyledons begin to pierce the seedbed surface) and continued across the VC (cotyledon stage where unifoliate leaves are unrolled sufficiently so that the leaf edges are not touching) and V1 (first node stage with fully developed leaves are present at unifoliate nodes) growth stages [32]. These stages were chosen as post-emergence damage of soybean due to pests mainly occur at these phases [37, 38].

The rates of post-emergence damage was calculated as described previously [37]:

$$Post-emergence\;damage\;(\%)=100\;X\frac{TDSdt}{TES}$$

Where TDSdt is the number of damaged seedlings per damage type (i.e. partially-damaged cotyledons, uprooted seedlings, or chewed seedlings or seedling parts) and TES is the total number of emerged seedlings. Arcsine and square root transformations were carried out on all percent data prior to apply statistical analysis to improve homogeneity of variance [39].

#### 2.2.7. Soybean biomass at wheat harvest

Another critical factor determining soybean productivity in wheat-soybean RC is the amount of biomass accumulated during the competition phase. A higher quantity of biomass may suggest a higher competition ability of soybean with wheat or a higher shade tolerance during this phase, which is an important indicator of grain yield. Soybean above-ground biomass for all varieties was quantified at wheat harvest by cutting the entire plants at the base along one linear meter on two rows (1 m^2^/micro-plot, 3 micro-plots in total/variety). The fresh biomass was dried in an oven at 80°C for 48h and weighed to obtain the dry weight expressed as g/m^2^.

#### 2.2.8. Statistical analyses

A two-way ANOVA followed by a Tukey’s HSD *post-hoc* test was performed to detect any significant effect of the year and cropping system on germination and emergence rates and vigor, causes of non-emergence, post-emergence damage and the biomass production at wheat harvest. We did not focus on wheat cultivar’s effect on soybean as our objective was to understand any potential differences among soybean cultivars independent of the primary crop over the two experimental years. When there was a significant effect, we separately analyzed data collected over the two experimental years using a one-way ANOVA followed by a Tukey’s HSD *post-hoc* to assess significant differences between soybean varieties and cropping systems. When no significant differences were found among soybean varieties in RC, all the data from RC were pooled to compare the mean difference with the data from CC. The Kruskal–Wallis non-parametrical test was used when the homogeneity of variances was not respected following data transformation. In this case, average values were compared using a Dunn test when the Kruskal–Wallis test was significant. All statistical analyses were applied using software R [40].

## 3. Results

### 3.1. Seed germination response to water potential under laboratory conditions

The response of seed germination rates of soybean cultivars to different water potentials and the base water potential value for each soybean cultivar are reported in **Table 2**. Average rates of seed germination were 98%, 98%, 95% and 84% at 0 MPa, -0.1 MPa, -0.25 MPa and -0.5 MPa, respectively while no germination occurred at -0.75 MPa. The tested soybean cultivars showed significant differences at -0.10 MPa (p<0.05), -0.25 MPa (p<0.001) and -0.5 MPa (p<0.001) while they showed no significant differences in germination rates at 0 MPa. Significant differences (p<0.001) in Ψ_b_ were found among the tested cultivars with the lowest (-0.65 MPa) and highest (-0.45 MPa) values for cv. ES Pallador and cv. RGT Speeda, respectively.

### 3.2. Sowing depth, seed germination dynamics and final rates

Results of sowing depth and seed germination are presented in **S1 Table**. Average sowing depth of cv. ES Pallador ranged from 4 to 5 cm and there were no significant differences between RC and CC. Likewise, average final germination rates under field conditions ranged from 96% to 100% with no significant differences between RC and CC. Completion of the seed germination phase took significantly longer time in RC compared to CC both in 2021 (6 vs. 4 days) and 2022 (5 vs. 3 days) **(Fig 1)**.

### 3.3. Variability in seedling emergence vigor, final emergence rates and their correlations between soybean cultivars and cropping systems

**Fig 2:**
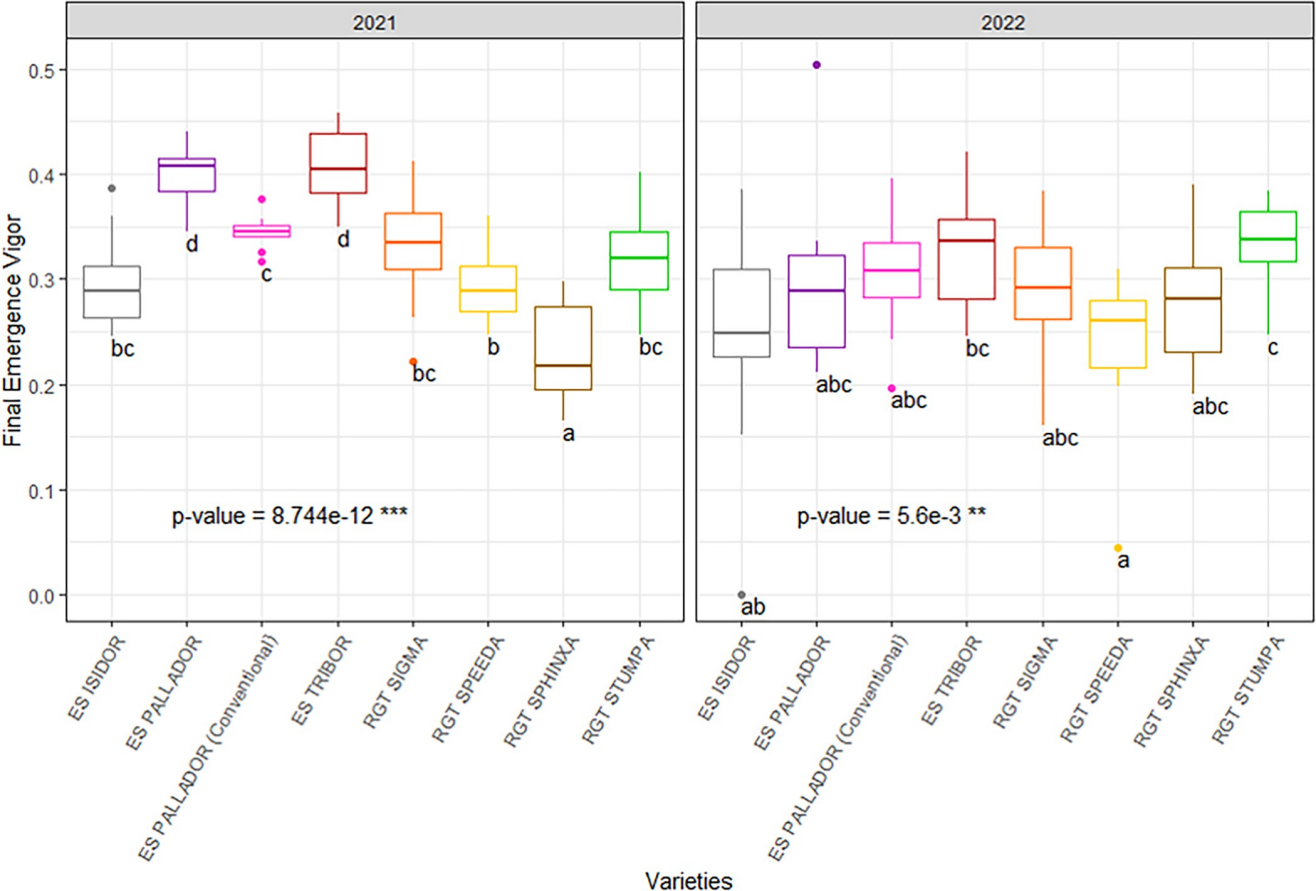
Seedling emergence vigor of seven soybean cultivars in wheat-soybean relay cropping systems over the two experimental years 2021–2022 at the Auzeville experimental station. Vertical bars reported in the figure represent confidence intervals. Details about cultivars are reported in Table 1.

Results concerning seedling emergence vigor under field conditions are reported in **Fig 2**. We found significant differences (p<0.001) among soybean cultivars with the values of SEVI ranging from 0.23 (for cv. RGT Sphinxa) to 0.41 (for cv. ES Tribor) in 2021 and from 0.24 (for cvs. ES Isidor and RGT Speeda) to 0.33 (for cvs. ES Tribor and RGT Stupa) in 2022. Significant effects of cultivar (p<0.001), year (p<0.001) and cultivar*year interactions (p<0.001) were observed on SEVI. SEVI was significantly higher in RC compared to CC in 2021 (0.40 vs. 0.32) while no significant difference was observed in 2022 between RC and CC with the values of 0.29 and 0.31, respectively. T_50_ values ranged from 10 to 14 DAS in 2021 (mean value 13) and from 8 to 10 DAS in 2022 (mean value 9; **S2 Table**). No correlation was found between T_50_ values and the final emergence vigor. Overall, SEVI was dependent on the seedling emergence dynamics **(S2 Fig)**.

**Fig 3:**
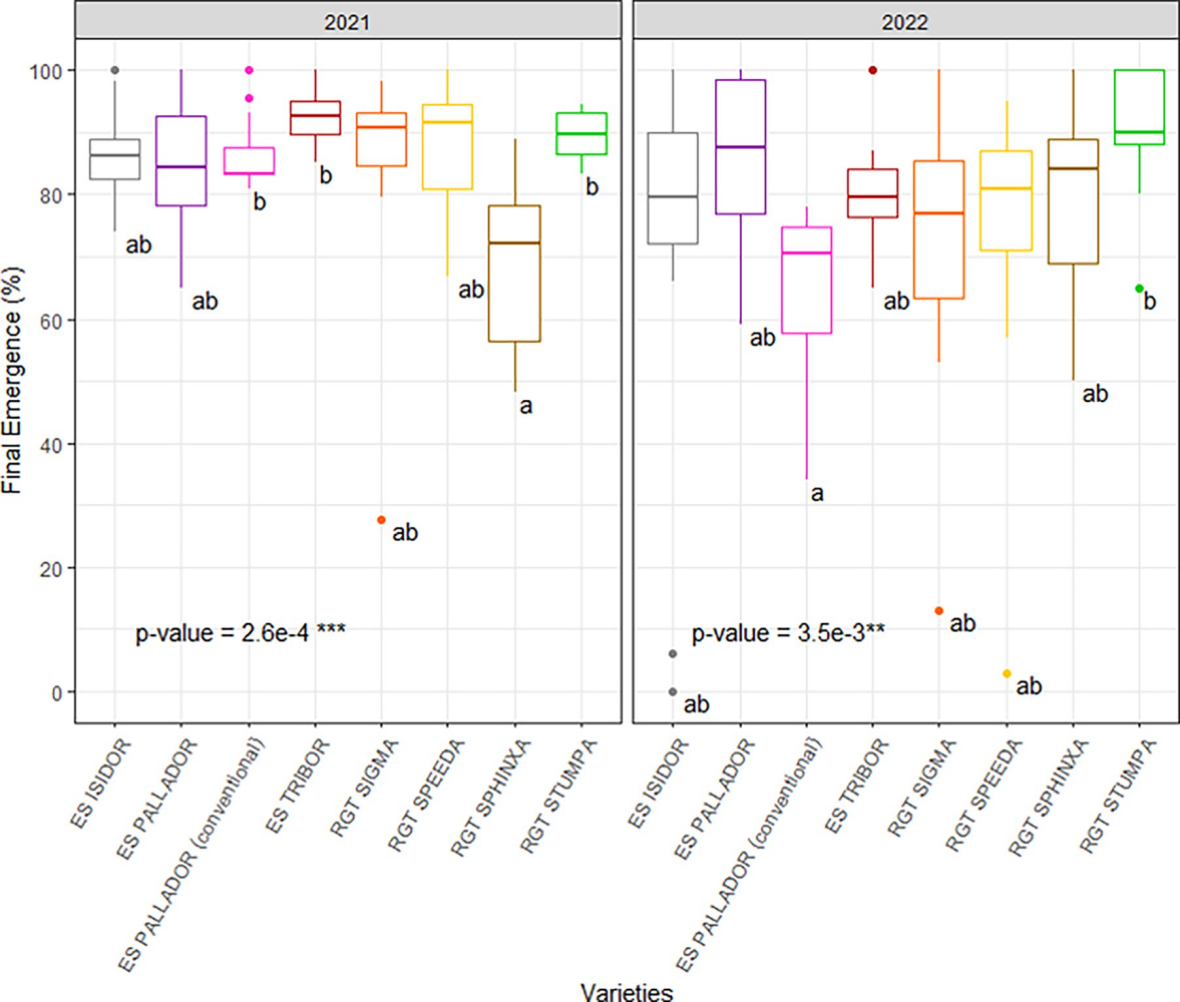
Final emergence rates of seven soybean cultivars in wheat-soybean relay cropping systems over the two experimental years 2021–2022 at the Auzeville experimental station. Vertical bars reported in the figure represent confidence intervals. Details about cultivars are reported in Table 1.

Results concerning final rates of seedling emergence under field conditions are reported in **Fig 3.** Final emergence rates ranged from 69% (for cv. RGT Sphinxa) to 93% (for cv. ES Tribor) in 2021 and from 70% (for cvs. ES Isidor) to 90% (for cv. RGT Stupa) in 2022. Significant effects of cultivar (p<0.01), year (p<0.05) and cultivar*year interactions (p<0.01) were observed on seedling final emergence rates. No significant difference in final emergence rates of cv. ES Pallador was observed between RC and CC which were 84% and 87% in 2021 and 85% and 65% in 2022, respectively. Seedling emergence of cv. ES Pallador took 22 days both in 2021 and 2022 **(Fig 3)**.

**Fig 4:**
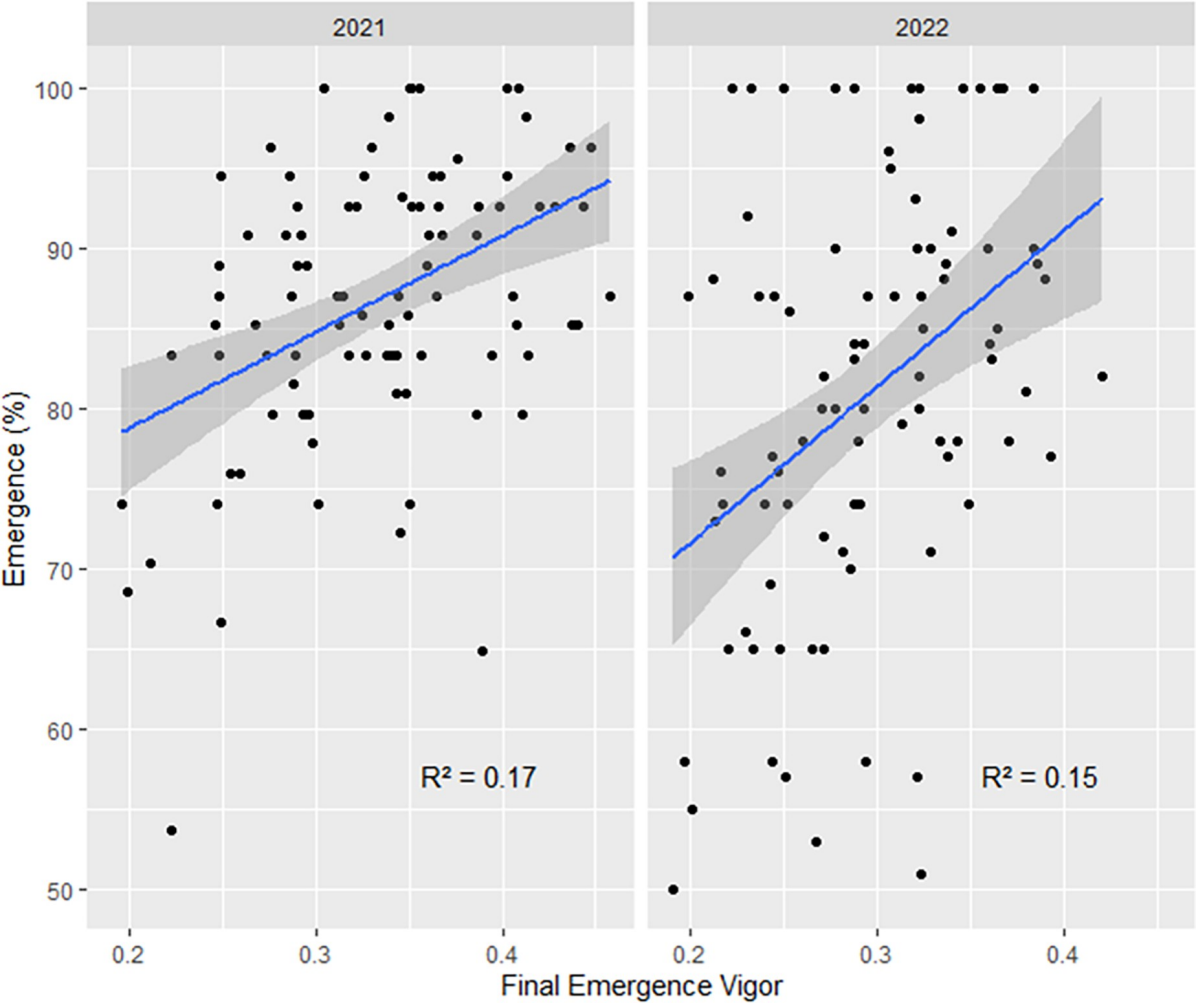
Correlation between final emergence rates and emergence vigors of seven soybean cultivars in wheat-soybean relay cropping systems over the two experimental years 2021–2022 at the Auzeville experimental station. Details about cultivars are reported in Table 1.

Results showing correlation between SEVI and final emergence rates are reported in **Fig 4**. Overall, we found that final emergence rates are positively correlated with SEVI with the coefficient of determination of 0.17 in 2021 and 0.15 in 2022.

### 3.4. Causes of non-emergence between soybean cultivars and cropping systems

Average rates of non-emergence and main causes that prevented seedlings from being emerged are reported in **S1 Fig**. In RC, the average rates of non-emergence ranged from 7% (for cv. ES Tribor) to 31% (for cv. RGT Sphinxa) in 2021 and from 10% (for cv. RGT Stumpa) to 30% (for cv. ES Isidor) in 2022. No significant differences in average emergence losses between RC and CC were observed in 2021 (16% and 13% in RC and CC, respectively) while emergence losses were significantly lower (p<0.01) in RC compared to those in CC (15% and 35% in RC and CC, respectively) in 2022. Overall, the presence of a soil surface crust and soil clods in the seedbed, seedling predation by pigeons and too deep sowing were the main causes of pre-emergence seedling mortality. Significant differences in pre-emergence seedling losses between RC and CC due to a soil surface crust (p<0.001) and to deep sowing (p<0.001 and p<0.05) was found both in 2021 and in 2022. In contrast, significant differences in pre-emergence seedling losses between RC and CC were found due to pigeons (0% and 17% in RC and CC, respectively; p<0.001) and soil clods (0% and 8% in RC and CC, respectively; p<0.05) only in 2022.

### 3.5. Causes of post-emergence damage between soybean cultivars and cropping systems

**Fig 5:**
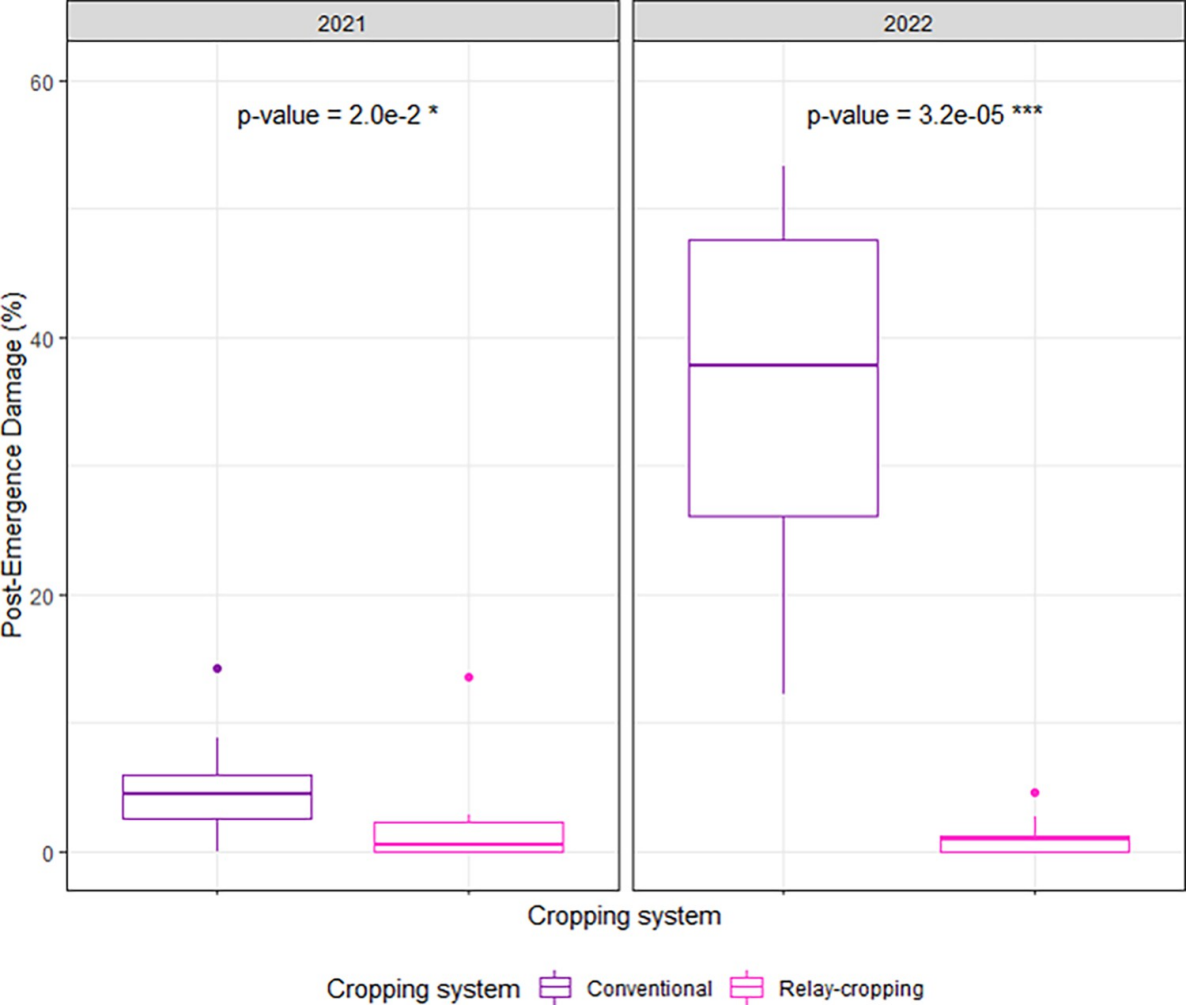
Difference in post-emergence seedling losses due to pigeons between relay- and conventional-cropped soybean over the two experimental years 2021–2022 at the Auzeville experimental station. Results on relay cropping are based on the pooled data on seven soybean cultivars, as there was no significant effect of soybean cultivar on pre-emergence losses, while data on conventional cropping is based only on cv. ES Pallador. Vertical bars reported in the figure represent confidence intervals. Details about cultivars are reported in Table 1.

The average rates of post-emergence losses of young soybean seedlings are reported in **Fig 5**. The common wood pigeon (*Columba palumbus*) was the only cause leading to post-emergence damage of soybean seedlings that consisted of either partial damage on the cotyledons as soon as they began to pierce from the seedbed surface or uprooting of seedlings from the seedbed. No significant effect of soybean cultivars on pre-emergence losses was found in RC and consequently the data on seven soybean cultivars were pooled and analyzed to compare with post-emergence losses of cv. ES Pallador grown in CC. Post-emergence losses in RC were significantly lower from those occurred in CC both in 2021 (2% and 5% in RC and CC, respectively; p<0.05) and 2022 (1% and 37% in RC and CC, respectively; p<0.001).

### 3.6. Variability in soybean biomass at wheat harvest between soybean cultivars and cropping systems

The average dry biomass values of seven soybean varieties at harvest are reported in **Table 4**. The duration of the competition phase was 63 days in 2021 and 71 days in 2022. In RC, the mean biomass values ranged from 23 to 45 g/m^2^ in 2021 and from 54 to 69 g/m2 in 2022. No statistical differences were found between the soybean varieties in RC in terms of their potential biomass production at wheat harvest. In contrast, there were significant differences between RC and CC with significantly higher biomass production in CC compared to that in RC both in 2021 (162 vs 33 g/m^2^; p<0.001) and 2022 (252 vs 60 g/m^2^; p<0.001).

**Table 4:** Average dry biomass (g/m^2^± SD) of seven soybean cultivars at wheat harvest under relay cropping for two consecutive years 2021–2022 at the Auzeville experimental station.

Cultivar	Dry biomass (g/m^2^)^#^	
	2021	2022
RGT SIGMA	43^a^ ± 16	62^a^ ± 23
RGT SPHINXA	20 ^a^ ± 9	69 ^a^ ± 7
RGT STUMPA	39 ^a^ ± 15	66 ^a^ ± 8
ES TRIBOR	45 ^a^ ± 5	60 ^a^ ± 16
RGT SPEEDA	36 ^a^ ± 9	56 ^a^ ± 21
ES ISIDOR	28 ^a^ ± 3	54 ^a^ ± 28
ES PALLADOR Relay	23 ^a^ ± 1	55 ^a^ ± 14
ES PALLADOR Conventional	162 ^b^ ± 22	252 ^b^ ± 41
**Significance level**	***	***
**Mean Relay cropping**	**33** ^**a**^ **± 12**	**60** ^**a**^ **± 16**
**Mean conventional cropping**	**162** ^**b**^ **± 22**	**252** ^**b**^ **± 41**
**Significance level**	***	***

^#^The duration of the competition phase was 63 days in 2021 and 71 days in 2022.

Means followed by the same letter are not significantly different at p < 0.05

***p<0.001

## 4. Discussion

### 4.1. Water limitation in the seedbed as the key factor affecting soybean germination and emergence under relay cropping

We observed that water stress in the seedbed is the most important limiting factor affecting soybean establishment in RC to a much greater extent than in CC. Indeed, during the seed germination phase, despite 26 mm of cumulated rainfall in 2021 from sowing until 6 DAS, water stress threshold in the seedbed for soybean germination was higher than the gravimetric water content at 0–10 cm in the seedbed (13% and 11%, respectively). This could be the key reason for which the completion of seed germination was slow in RC (6 DAS ~90°Cd) compared to that in CC (4 DAS ~60°Cd). However, during the seedling emergence phase, water stress threshold in 2021 was lower compared to the gravimetric water content at 0–10 cm thanks to frequent rainfall that occurred after the completion of soybean germination (50 mm of cumulated rainfall post-germination until the completion of emergence). In contrast, water stress in the seedbed was much higher in 2022 as almost no rainfall (only 4 mm) occurred from sowing until the completion of emergence of soybean requiring an application of irrigation (35mm at 3 DAS) to ensure seed germination and emergence. Despite this irrigation, water stress in the seedbed occurred as the gravimetric water content at 0–10 cm in the seedbed under RC was much lower compared to water stress threshold in the seedbed from 7 DAS until the completion of emergence. This was probably the reason for which despite a much higher average air temperature in 2022 compared to that of 2021 (14±1°C in 2021 vs. 22±2°C in 2022) it took the same time (22 DAS) for soybean in both years to attain the maximum emergence rates.

We found that the average T_50_ value was much higher in 2021 compared to that in 2022 (13 vs. 9 DAS). This indicates that stress factors, especially the seedbed moisture, were more pronounced in 2021 compared to 2022. Indeed, the total quantity of water (rainfall, irrigation or both) applied to soybean in 2021 from sowing until the completion of emergence was 76 mm vs. 95 mm in 2022 while the average gravimetric moisture content at 0–10 cm during the same period was 13% in 2021 vs. 14% in 2022. This clearly shows that seedbed moisture is the most important abiotic factor affecting soybean seedling emergence dynamics under RC across southern France. This is further confirmed by the absence of correlation between T_50_ values and the final emergence vigor indicating that seedling emergence dynamics is strictly dependent on stress factors they encounter during emergence.

Despite different tolerance of the seven soybean cultivars to water stress, as shown by different Ψ_b_ values, under laboratory conditions, no correlation between Ψ_b_ values and emergence vigor of soybean cultivars was detected under field conditions. This was probably because frequent rainfall in 2021 and the irrigation application in 2022 after sowing reduced water stress in the seedbed thereby masking the water stress tolerance of soybean varieties. On the other hand, avoiding irrigation after sowing in the absence of rainfall, as that occurred in 2022, is too risky as this may lead to germination and emergence failure under field conditions. Future studies under controlled environmental conditions with different water content regimes of soil may clarify whether soybean varieties with a much lower Ψ_b_ values may have higher germination and emergence vigor in soil with lower moisture availability.

### 4.2. Effect of the primary crop on soybean establishment and biomass production during the competition phase

We used two different wheat cultivars (a soft and a durum wheat) and two different sowing dates (winter vs. spring) over the 2-year study. Although each wheat genotype may have its own characteristics including the plant architecture and competitive ability for resources, we did not measure potential effects of wheat cultivars and the sowing dates on soybean establishment and biomass production during the competition phase. More specifically to crop establishment phase, competition for water between wheat and soybean was the most important factor compared to that for light and nutrients as the latter competition start sonly during the autotrophic phase (i.e. post-emergence). Indeed, seedlings developing during heterotrophic growth phase are not able to perform photosynthetic activity and they exclusively use carbon sources for growth from organic compounds stored in the seed [41]. Consequently, the competition between the two crops during the intercropping period begins only when seedlings acquire photosynthetic competence, following heterotrophic-to-autotrophic transition after seedling establishment.

In contrast to soybean establishment phase, we observed an important effect of the competition phase on soybean biomass as shown by the data measured at wheat harvest. There was a 2-fold difference in dry soybean biomass at wheat harvest with much lower average in 2021 (33 g/m^2^) compared to that of 2022 (60 g/m^2^) that could be due to an increased competition of wheat for resources towards soybean (e.g. water, light, nutrients etc.). Indeed, soft wheat was sown much earlier for the RC trial in 2021 (on 10 November 2020) compared to durum wheat sown much later for the RC trial in 2022 (on 3 February 2022). That would have allowed higher growth and development of wheat in 2021 with an increased above- (e.g. height and biomass production) and below-ground biomass (root development) with much higher competition with soybean which instead was planted on similar dates in both years (i.e. 6 May in 2021 and 9 May in 2022).

### 4.3. Relay cropping as a more water reliant system than sole cropping?

We found that the gravimetric water content at 0-10cm soil depth under irrigated RC and non-irrigated CC were very similar. For instance, RC and CC plots were not irrigated in 2021 until the completion of emergence but the daily mean values of the gravimetric water content at 0-10cm soil depth under CC was slightly higher than those under RC **(Fig 1)**. In contrast, in 2022, despite 38 mm of water applied to RC plots at 3 DAS, the daily mean values of the gravimetric water content at 0-10cm soil depth under CC was much higher than those under RC (except 4 days following irrigation) which can be explained by two factors. First, the presence of wheat with much higher height and biomass compared to soybean during soybean establishment phase catches hold of water preventing the droplets to fall down and moisten the seedbed surface (physical barrier to water access to the seedbed), especially when rainfall is not intense. Second, due to advanced growth phase and well-developed below ground biomass (i.e. roots), wheat rapidly absorbs the moisture especially when there is limited rainfall or irrigation (i.e. wheat is more competitive than soybean for water use due to the difference in growth stage). These are the reasons for which soybean as a relay crop in wheat-soybean RC takes longer time to seed imbibition and germination compared to soybean sown under CC. A 4- to 5-fold higher soybean biomass under CC compared to that under RC in both years further suggests how early soybean development is affected by competition for resources between wheat and soybean that may have an important impact on soybean productivity. All this suggests that a successful soybean seedling emergence under RC is strictly dependent on the water input and that early irrigation applications are important in the absence of sufficient rainfall that allows to increase the gravimetric water content of the seedbed at a higher level than the water stress threshold for soybean germination. A higher frequency of drought and heat waves are expected in the near future across Europe [42] and increased drought events could affect soybean production especially across southern Europe [43, 44] whereby soybean production will be increasingly dependent on supplementary irrigation [45]. Therefore, farmers willing to practice RC of soybean across southern France should be equipped with the possibility of irrigation both before and after soybean planting in RC.

### 4.4. Relay cropping as an effective planting method for limiting bird damage to sprouting soybean seedlings?

Damage of sprouting soybean seedlings due to birds may lead to severe losses affecting soybean establishment under CC. A study from India showed that doves and pigeons may cause up to 80% losses from germination up to the green cotyledon stage [38]. Another study in Southern France based on field observation reported wood pigeons and wild hares as the most important biotic stresses affecting sprouting soybean seedlings, especially during and after emergence with up to 50% of damage [37]. However, the extent of damage at the field plot scale seems to be highly variable depending on the sowing date, the composition of crop species in neighboring plots, the type of landscape surrounding the field plots including the distance of the experimental site from the residential area and semi-natural habitats (e.g. hedges). Here we observed wood pigeon as the most important vertebrate pest affecting soybean seedlings both pre- and post-emergence although the damage was significantly lower under RC compared to CC. It is possible that the presence of wheat at an advanced growth stage acted as physical barrier affecting the movements and feeding behavior of wood pigeons that needs further exploration. The much higher seedling damage due to wood pigeons in 2022 under CC compared to that occurred in 2021 can be explained by the fact that experimental plots in 2022 were much closer to residential areas (~200 m) compared to those in 2021 (~450 m).

## 5. Conclusion

To the best of our knowledge, this is the first study in Europe assessing genetic variability in early growth traits of soybean cultivars under wheat-soybean RC by comparing also to CC. We found significant differences among soybean cultivars in early growth traits although their responses seem to be affected by seedbed conditions, as shown by inconsistent responses over the two experimental years. We also showed an important contrast in seedbed moisture conditions between RC and CC and how this affects soybean establishment and initial growth during the competition phase. Finally, we found that RC may significantly reduce vertebrate pests damage to sprouting soybean seedlings compared to CC. Future studies are however needed to assess a higher number of soybean cultivars with contrasted Ψ_b_ values to understand whether soybean varieties characterized by lower Ψ_b_ values (i.e. higher water stress tolerance) can perform better under field conditions using different irrigation regimes. This information may help farmers in their decision making about the best varieties to be used for an improved soybean establishment and early growth.

## Supporting information

S1 Fig Difference in pre-emergence seedling losses between relay- and conventional-cropped soybean over the two experimental years 2021–2022 at the Auzeville experimental station. Results on relay cropping are based on the pooled data on seven soybean cultivars, as there was no significant effect of soybean cultivar on pre-emergence losses, while data on conventional cropping is based only on cv. ES Pallador. Vertical bars reported in the figure represent confidence intervals. Details about cultivars are reported in Table 1.
(DOCX)

S2 Fig Correlation between time to 50% seedling emergence (T50) and final emergence vigor of soybean over the two experimental years 2021–2022 at the Auzeville experimental station. (DOCX)

S1 Table Sowing depths (cm ± SD) and final germination rates (% ± SD) of soybean cv. ES Pallador in relay and conventional cropping systems over the two experimental years 2021–2022.
(DOCX)

S2 Table Emergence dynamics of seven soybean cultivars in wheat-soybean relay cropping system over the two experimental years 2021–2022 at the Auzeville experimental station. Details about cultivars are reported in Table 1.
(DOCX)
