# Supplementary material for: Seedling emergence and biomass production of soybean cultivars under wheat-soybean relay cropping
Source: PLoS One. 2023 Nov 1;18(11):e0293671. doi: 10.1371/journal.pone.0293671 (PMC10619765; doi:10.1371/journal.pone.0293671)
Supplement: S1 Fig — Results on relay cropping are based on the pooled data on seven soybean cultivars, as there was no significant effect of soybean cultivar on pre-emergence losses, while data on conventional cropping is based only on cv. ES Pallador. Vertical bars reported in the figure represent confidence intervals. Details about cultivars are reported in Table 1. (DOCX) [file pone.0293671.s001.docx]

**Figure S1.** Difference in pre-emergence seedling losses between relay- and conventional-cropped soybean over the two experimental years 2021-2022 at the Auzeville experimental station. Results on relay cropping are based on the pooled data on seven soybean cultivars, as there was no significant effect of soybean cultivar on pre-emergence losses, while data on conventional cropping is based only on cv. ES Pallador. Vertical bars reported in the figure represent confidence intervals. Details about cultivars are reported in Table 1.

**
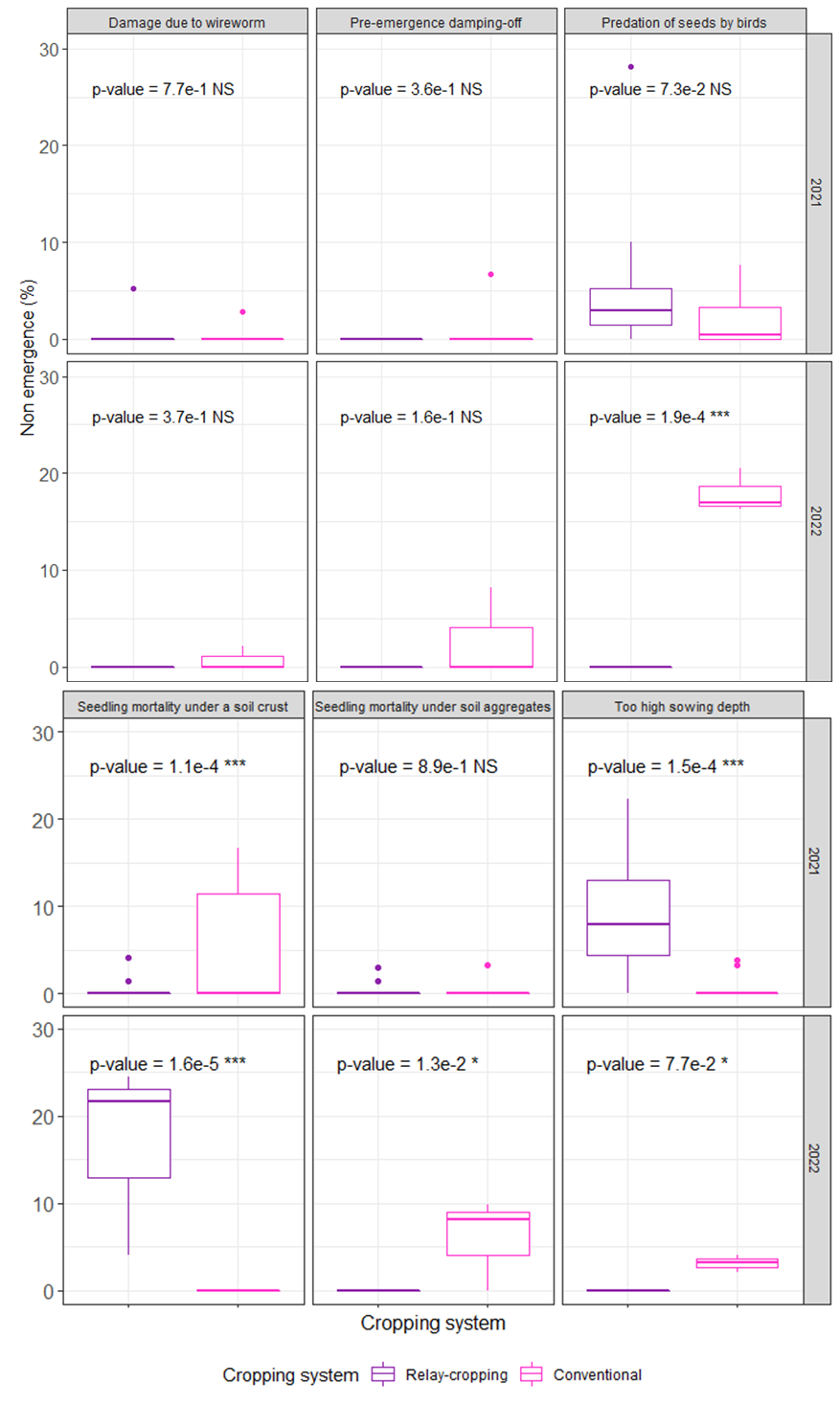
**
