# Supplementary material for: Seedling emergence and biomass production of soybean cultivars under wheat-soybean relay cropping
Source: PLoS One. 2023 Nov 1;18(11):e0293671. doi: 10.1371/journal.pone.0293671 (PMC10619765; doi:10.1371/journal.pone.0293671)
Supplement: S2 Fig — (DOCX) [file pone.0293671.s002.docx]

**Figure S2.** Correlation between time to 50% seedling emergence (T50) and final emergence vigor of soybean over the two experimental years 2021-2022 at the Auzeville experimental station.
