# Supplementary material for: Seedling emergence and biomass production of soybean cultivars under wheat-soybean relay cropping
Source: PLoS One. 2023 Nov 1;18(11):e0293671. doi: 10.1371/journal.pone.0293671 (PMC10619765; doi:10.1371/journal.pone.0293671)
Supplement: S1 Table — ES Pallador in relay and conventional cropping systems over the two experimental years 2021–2022. (DOCX) [file pone.0293671.s003.docx]

**Table S1.** Sowing depths (cm ± SD) and final germination rates (% ± SD) of soybean cv. ES Pallador in relay and conventional cropping systems over the two experimental years 2021-2022.

| **Cultivar/system** | **Sowing depth (cm)** | | **Final germination (%)** | |
| --- | --- | --- | --- | --- |
|  | **2021** | **2022** | **2021** | **2022** |
| ES PALLADOR Relay | 3.97 ± 0.25 | 4.67 ± 0.6 | 100 ± 0 | 98 ± 4 |
| ES PALLADOR Conventional | 4.18 ± 0.15 | 5.4 ± 0.2 | 100 ± 0 | 93 ± 5 |
| **Mean** | **4.07 ± 0.2** | **5.03 ± 0.4** | **100 ± 0** | **96 ± 4** |
| System | ns | | ns | |
| Year | ns | | ns | |
| System*Year | ns | | ns | |
| ns: not significant | | | | |
