# Supplementary material for: Seedling emergence and biomass production of soybean cultivars under wheat-soybean relay cropping
Source: PLoS One. 2023 Nov 1;18(11):e0293671. doi: 10.1371/journal.pone.0293671 (PMC10619765; doi:10.1371/journal.pone.0293671)
Supplement: S2 Table — Details about cultivars are reported in Table 1. (DOCX) [file pone.0293671.s004.docx]

**Table S2.**  Emergence dynamics of seven soybean cultivars in wheat-soybean relay cropping system over the two experimental years 2021-2022 at the Auzeville experimental station. Details about cultivars are reported in Table 1.

| **Cultivar** |  | **Emergence (%) days after sowing (DAS) in 2021** | | | | | |  | **T_50_ (DAS)** |
| --- | --- | --- | --- | --- | --- | --- | --- | --- | --- |
|  |  | **9** | **11** | **12** | **13** | **15** | **22** |  |  |
| RGT SIGMA |  | 1^b^ ± 0 | 26^cd^ ± 12 | 43^cde^ ± 14 | 63^bc^ ± 15 | 78^b^ ± 11 | 85^ab^ ± 11 |  | 13 |
| RGT SPHINXA |  | 0^b^ ± 0 | 4^e^ ± 3 | 13^f^ ± 8 | 24^d^ ± 13 | 49^c^ ± 13 | 69^b^ ± 8 |  | 17 |
| RGT STUMPA |  | 7^b^ ± 0 | 15^cd^ ± 9 | 31^cde^ ± 13 | 54^abc^ ± 11 | 74^ab^ ± 5 | 89^b^ ± 2 |  | 14 |
| ES TRIBOR |  | 7^a^ ± 0 | 58^a^ ± 9 | 74^a^ ± 7 | 82^a^ ± 4 | 88^ab^ ± 3 | 93^a^ ± 3 |  | 10 |
| RGT SPEEDA |  | 0^b^ ± 0 | 13^de^ ± 6 | 32^de^ ± 11 | 57^c^ ± 12 | 77^b^ ± 8 | 88^ab^ ± 6 |  | 14 |
| ES ISIDOR |  | 1^b^ ± 0 | 15^cde^ ± 8 | 31^e^ ± 9 | 54^c^ ± 11 | 74^b^ ± 8 | 86^ab^ ± 4 |  | 14 |
| ES PALLADOR Relay |  | 9^a^ ± 0 | 46^ab^ ± 10 | 61^ab^ ± 9 | 76^ab^ ± 8 | 81^ab^ ± 7 | 84^ab^ ± 6 |  | 12 |
| ES PALLADOR Conventional |  | 0^b^ ± 0 | 28^bc^ ± 6 | 49^bcd^ ± 5 | 71^abc^ ± 6 | 80^b^ ± 5 | 87^a^ ± 4 |  | 13 |
| **Mean** |  | **3 ± 2** | **25 ± 8** | **61 ± 10** | **62 ± 10** | **77 ± 7** | **85 ± 5** |  | **13 ± 1** |
|  |  | ******* | ******* | ******* | ******* | ******* | ****** |  |  |
| **Cultivar** |  | **Emergence (%) days after sowing (DAS) in 2022** | | | | | |  | **T_50_ (DAS)** |
|  |  | **7** | **8** | **9** | **11** | **22** |  |  |  |
| RGT SIGMA |  | 8^ab^ ± 9 | 40^abc^ ± 14 | 59^ab^ ± 16 | 68^ab^ ± 15 | 72^ab^ ± 15 |  |  | 10 |
| RGT SPHINXA |  | 8 ^ab^ ± 7 | 32 ^bca^ ± 15 | 55 ^ab^ ± 14 | 78 ^ab^ ± 11 | 80^ab^ ± 11 |  |  | 10 |
| RGT STUMPA |  | 14 ^ab^ ± 8 | 58 ^a^ ± 11 | 80 ^b^ ± 9 | 87 ^b^ ± 8 | 90^b^ ± 7 |  |  | 8 |
| ES TRIBOR |  | 17 ^ab^ ± 11 | 47 ^ab^ ± 12 | 68 ^ab^ ± 6 | 76 ^ab^ ± 6 | 81^ab^ ± 7 |  |  | 9 |
| RGT SPEEDA |  | 3 ^b^ ± 2 | 21^c^ ± 12 | 55 ^ab^ ± 15 | 70 ^ab^ ± 16 | 73^ab^ ± 16 |  |  | 10 |
| RGT ISIDOR |  | 6 ^b^ ± 6 | 24 ^bc^ ± 16 | 58 ^ab^ ± 19 | 67 ^ab^ ± 21 | 70^ab^ ± 22 |  |  | 10 |
| ES PALLADOR Relay |  | 8 ^ab^ ± 6 | 33 ^abc^ ± 14 | 71 ^ab^ ± 10 | 83 ^ab^ ± 8 | 85^ab^ ± 9 |  |  | 9 |
| ES PALLADOR Conventional |  | 22 ^a^ ± 7 | 39 ^abc^ ± 10 | 52 ^a^ ± 9 | 65 ^a^ ± 9 | 65^a^ ± 9 |  |  | 10 |
| **Mean** |  | **12 ± 8** | **39 ± 12** | **63 ± 10** | **75 ± 10** | **77 ± 10** |  |  | **9 ± 1** |
|  |  | ****** | ******* | ****** | ****** | ****** |  |  |  |
| Means followed by the same letter are not significantly different at p < 0.05; ***p < 0.001; **p < 0.01 | | | | | | | |  |  |
